# ELTD1—An Emerging Silent Actor in Cancer Drama Play

**DOI:** 10.3390/ijms22105151

**Published:** 2021-05-13

**Authors:** Ani-Simona Sevastre, Iuliana M. Buzatu, Carina Baloi, Alexandru Oprita, Alexandra Dragoi, Ligia G. Tataranu, Oana Alexandru, Stefania Tudorache, Anica Dricu

**Affiliations:** 1 Department of Pharmaceutical Technology, Faculty of Pharmacy, University of Medicine and Pharmacy, 200349 Craiova, Romania; anifetea_umf@yahoo.com; 2 Department of Biochemistry, Faculty of Medicine, University of Medicine and Pharmacy, 200349 Craiova, Romania; buzatu_iuliana@yahoo.com (I.M.B.); carina_baloi@yahoo.com (C.B.); opr.alexandru@gmail.com (A.O.); alexa.dragoi@yahoo.com (A.D.); anica.dricu@live.co.uk (A.D.); 3 Department of Neurosurgery, Bagdasar-Arseni Hospital, 041915 Bucharest, Romania; ttranu@gmail.com; 4 Department of Neurology, Faculty of Medicine, University of Medicine and Pharmacy, 200349 Craiova, Romania; oanale@hotmail.com; 5 Department of Obstetrics and Gynecology, Faculty of Medicine, University of Medicine and Pharmacy, 200349 Craiova, Romania

**Keywords:** ELTD1, biomarker, angiogenesis, cancer

## Abstract

The epidermal growth factor, latrophilin, and seven transmembrane domain–containing protein 1 (ELTD1), is a member of the G–protein coupled receptors (GPCRs) superfamily. Although discovered in 2001, ELTD1 has been investigated only by a few research groups, and important data about its role in normal and tumor cells is still missing. Even though its functions and structure are not yet fully understood, recent studies show that ELTD1 has a role in both physiological and pathological angiogenesis, and it appears to be a very important biomarker and a molecular target in cancer diseases. Upregulation of ELTD1 in malignant cells has been reported, and correlated with poor cancer prognosis. This review article aims to compile the existing data and to discuss the current knowledge on ELTD1 structure and signaling, and its role in physiological and neoplastic conditions.

## 1. Introduction

With major implications in cancer, the tyrosine kinases receptors (RTKs) are one of the most analyzed and reviewed research topics [1,2,3,4,5,6,7]. New ways of activating and transmitting the intracellular signal involved in cancer are constantly being discovered. Data indicates that one route of RTKs activation may be through agonists of G protein–coupled receptors (GPCRs) [8,9,10]. This phenomenon is called transactivation, and is considered to be an important pathway, involved in growth–promoting activity of GPCR ligands [11].

Comprising over 900 members and with more than 2% of the genes encoded by the human genome, the GPCRs family is by far the largest family of cell–surface signaling molecules. Of ancient origin, the adhesion GPCRs seem to have had a role in allowing cells to adhere and intercommunicate during the metazoan multicellularity evolution [12]. The GPCRs are involved in the control of the most important physiological functions, such as: neurotransmission, immune response, hormone and enzyme release, and contraction of smooth and cardiac muscles, with at least 15 of their receptors being dysregulated in a wide range of human chronic diseases, especially in tumors [13,14].

Discovered in developing cardiomyocytes, the epidermal growth factor, latrophilin, and seven transmembrane domain–containing protein on chromosome 1 (ELTD1), also known as the adhesion G protein–coupled receptor L4 (ADGRL4), is a member of the GPCR superfamily [15] and one of the 33 members of the “adhesion family” [16], characterized by specific large extracellular domains with adhesion items, absent in other GPCR families [17].

It has been shown that, amongst other functions, ELTD1 regulates brain angiogenesis and promotes tumor growth and metastasis [18]. Furthermore, its expression in normal vasculature was found to be regulated by two angiogenic pathways: increased by vascular endothelial growth factor (VEGF) pathway and repressed by Delta–like ligand 4 (DLL4) from NOTCH intercellular signaling pathway [18,19,20]. Since its discovery, ELTD1 was associated with cardiac and renal function, glioblastoma, and colorectal cancer [18,21,22,23,24,25].

Clinical application of the therapies involving VEGF and NOTCH signaling pathways proved to be unsuccessful. Despite the broad antitumoral spectrum of VEGF inhibition and the preclinical optimistic results, implementing these strategies into clinical studies did not improve overall survival(OS), possibly due to tumor resistance [26]. It can be hypothesized that in time, tumors subjected to selective antiangiogenic treatments may be able to activate alternative parallel angiogenic pathways [27]. Therefore, such pathways represent important topics for current research and ELTD1 is a promising drug target. Several research groups published data from experiments using polyclonal (pAb) and monoclonal (mAb) antibody against ELTD1, showing that these novel treatment strategies may have high potential in glioma preclinical mouse models [28,29,30]. Furthermore, an increased level of cytotoxicity in glioblastoma cell lines could be achieved by silencing ELTD1 via siRNA [31,32]. Yet, little is known about ELTD1 functions and mechanisms of action. This review aims to summarize the current knowledge regarding ELTD1 and to highlight its importance as a possible candidate to be a part of an innovative therapeutic strategy, either alone or in combination with other conventional approaches already in use.

## 2. Roles

The ELTD1 receptor was discovered by Nechiporuk and colleagues using murine models, and it was found to be highly expressed in cardiomyocytes, blood vessels, and bronchi’s smooth muscle cells [15].

**Figure 1:**
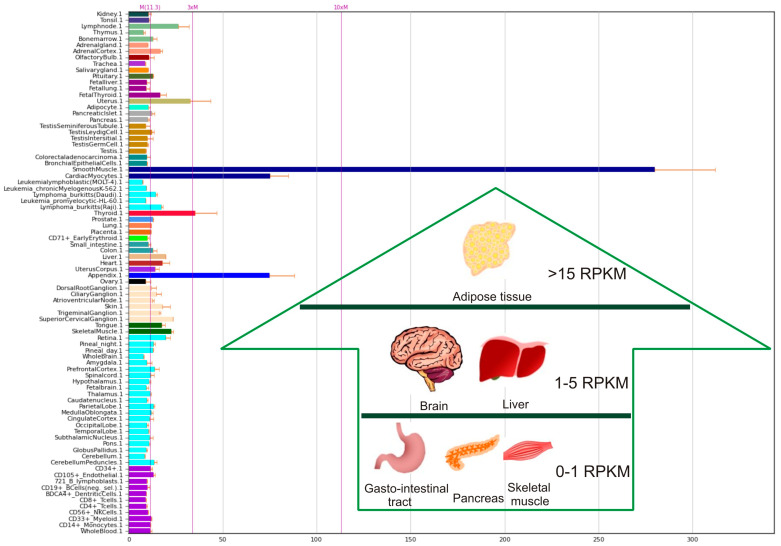
ELTD1 distribution in tissues. The results were retrieved from Genomics Biogps portal database [35]. Selective data regarding the distribution in the main organs was obtained from published RNAseq analysis [34]. Abbreviations: RPKM—reads per kilobase million.

Recently, Olaniru and colleagues studied the distribution of adhesion G–protein coupled receptors in human tissues [33]. The ELTD1 is highly distributed in some tissues, such as: adipose tissue, brain, liver, skeletal muscle, gastrointestinal tract, and pancreas [34,35] The distribution in different types of tissues may be observed in Figure 1, generated by accessing the Biogps portal database [35].

The GPCR superfamily of ELTD1 influences different processes, like: smell, taste, vision, chemotaxis, hormone secretion, and inflammation [36,37]. The orphan ELTD1 receptor was reported to be involved in angiogenesis [18,22,24,38] and cardiac hypertrophyin rat [21], sensitivity of anesthetics [39], subcutaneous fat thickness in pig [40], and tick burden in cattle [41].

ELTD1 involvement in various pathologies was also investigated. Studies revealed its upregulation in malignancies, such as: renal, colorectal, head, neck, and ovarian cancers [18].

Wallgard and colleagues suggested that ELTD1 is an important marker of microvascular endothelium in malignant diseases [38] and Towner et al. found that it is a valuable tumor marker in cases of glioma [42]. Moreover, Dai and colleagues proved that tumor progression may be inhibited by miR–139–5p, via targeting ELTD1 [43]. Then, in 2017 Ziegler and colleagues targeted ELTD1 for its antiangiogenic effect in glioma xenograft models [25]. In the same year, Serban F. and colleagues showed that by silencing of ELTD1, cell death may occur in glioblastoma cell lines [31].

Several studies positively associated the upregulation of ELTD1 on chromosome 1 in malignant cells with poor cancer prognosis. In 2019, a study reported that ELTD1 facilitates proliferation, migration, and invasion in glioma by activating the signaling axis JAK/STAT3/HIF–1α. Finally, high levels of ELTD1 were correlated with poor prognosis in human glioma in another study [44]. This data suggests that ELTD1 may be a potential target for prevention and treatment of glioma. However, the implication of ELTD1 in cancer behavior still remains mostly unknown.

## 3. Digging into Knowing the ELTD1 Mechanism

First discovered in 2001, ELTD1 expression has been shown in cardiomyocytes, vascular and bronchiolar smooth muscle cells (SMCs) in rat heart and lungs, and it has been suspected to be involved in cardiomyocyte differentiation and coronary angiogenesis [15].

Later, in 2008, Wallgard et al. found that ELTD1 mRNA was a broad marker for vascular endothelial cells in mouse [38]. Porto and colleagues proved that ELTD1 gene DNA variation was associated with tick burden in cattle [41]. In the same year, Lee at al suggested that ELTD1 gene is one of the eight neuronal genes influencing subcutaneous fat thickness in humans and pigs [40].

In 2013, another research group led by Towner et al. introduced the idea of “ELTD1 as novel biomarker for glioma” [42], and their studies were later expanded by others, then Xiao and colleagues linked the cardiac hypertrophy to the low ELTD1 expression in mice [21]. By microsatellite scanning of the immunogenome in transplantation of hematopoietic stem cell, Harkensee associated the ELTD1 and MAPK14 with graft–versus–host disease [45].

Simultaneously, by the assiduous work of Masiero et al., the endothelial orphan receptor ELTD1 was identified as an important regulator of angiogenesis [18]. Based on this discovery, other studies followed and generated valuable data and interesting hypothesis [24].

In 2015, Carty et al. conducted a meta–analysis of genome–wide association and identified ELTD1 as one of the genetic risk factors for stroke in the population of African Americans [46].

In the same year, Ziegler and colleagues begun the adventure in developing new therapies based on ELTD1 against mouse glioma models [47] followed by other studies that independently proved the role of this receptor, as molecular target, in glioma therapy [20,25].

In 2017, Favara et al. reported that ELTD1 is upregulated in breast cancer endothelium that in turn induced lipid metabolism downregulation [48] and later, in 2019, the same group demonstrated that ELTD1 silencing alters the cell metabolic profile in endothelial cells [32].

A year later, Kan and colleagues proved that the tumor microenvironment is regulating ELTD1 function in hepatocellular carcinoma [49].

Treatments targeting ELTD1 started to be investigated by several research groups. Zalles et al., provided some options for glioblastoma treatment using monoclonal antibodies and scFvantibody fragment, in a G55 xenograft mouse model [29,30]. One of the most recent research conducted by Niivirta et al. provided data regarding ELTD1, as a predictive marker for the treatment of renal cancer patients. Their results identified ELTD1 expression in tumor vessels as a positive predictive marker for sunitinib–treatment in metastatic renal cell cancer patients [22].

In the same time, the research area regarding this receptor expanded to other pathologies. For example, based on the fact that ELTD1 has been found to be associated to cannabis use disorder [50], Zhang and colleagues suggested a strong association of this receptor with schizophrenia [51].

By using cell lines and orthotopic xenograft mouse model, Santiago and colleagues recently demonstrated that ELTD1 is a potential target in retinoblastoma. They found that, without affecting normal cell viability, cell migration, and metastasis were reduced by ELTD1 disruption [52].

**Figure 2:**
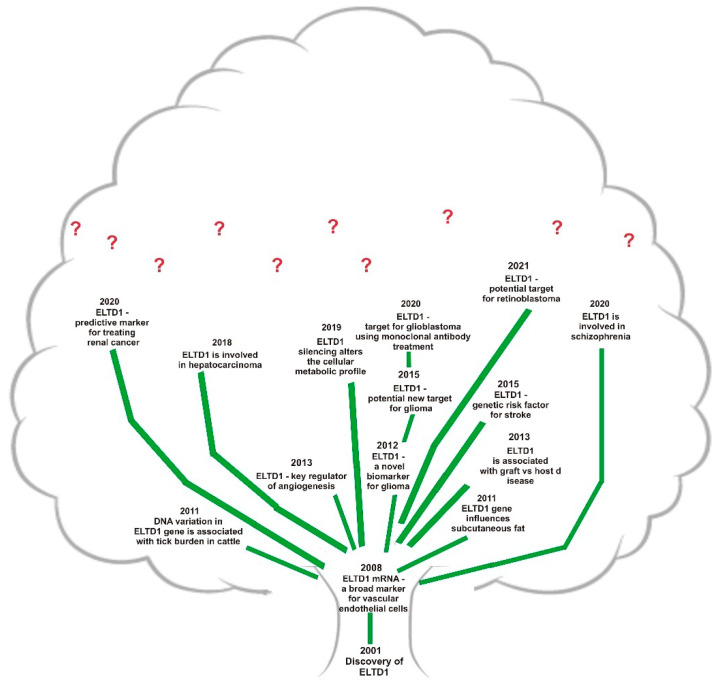
Branching the ELTD1 knowledge.

The most important studies involving ELTD1 are organized in chronological order in the Figure 2.

### 3.1. Structure and Signaling

ELTD1 (ADGRL4) is a member of the GPCR big family of receptors, which contains more than 900 members divided into five families: glutamate family, rhodopsin family, adhesion family, frizzled family, and secretin family [53].

Initially, the “adhesion family” was a part of the secretin family, but later, due to distinct characteristics, such as unusually elongated N–terminal ectodomain with adhesion–linked motifs [54], it was created as a distinct family. The orphan ELTD1 receptor and the 1, 2, and 3 latrophilin receptors are the fourmembers of the adhesion family, grouped in the latrophilin–like subfamily [17].

**Figure 3:**
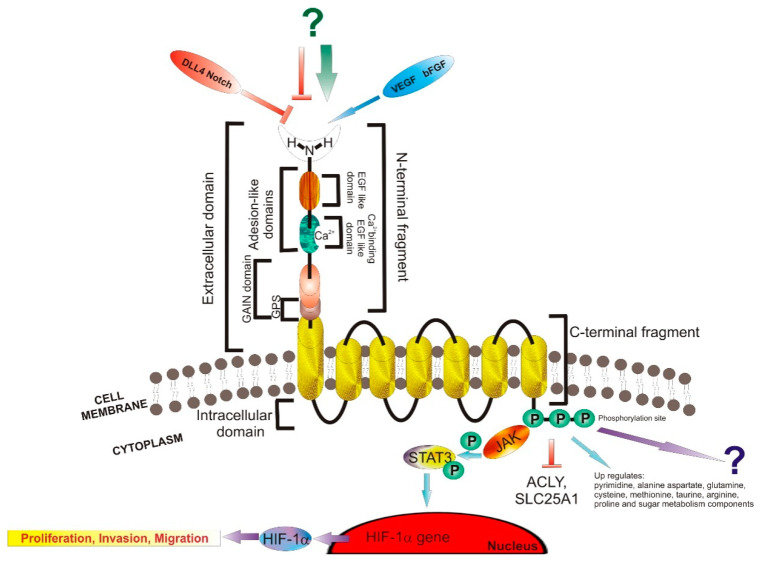
Structure of ELTD1. Abbreviations: VEGF—vascular epithelial growth factor, bFGF—fibroblast growth factor, DLL4—delta like ligand 4, Notch–GPS—G–protein coupled protein hormone receptor proteolysis site, EGF—epidermal growth factor, GAIN domain—G–protein–coupled receptor (GPCR) autoproteolysis–inducing domain. P—phosphate, JAK—Janus kinases, STAT3—signal transducer and activator of transcription protein 3, HIF–1α—Hypoxia–inducible factor 1–alpha, point arrow—activation, block arrow—inhibition.

Topographically, the ELTD1 receptor consists of an intracellular domain (ICD), a 7–trans–membrane domain (7TMD) and an extracellular domain (ECD) composed of an epidermal growth factor(EGF) domain, an EGF Ca2+ binding domain and a GPCR autoproteolysis site [15,27]. The adhesion motifs are represented by the EGF domain and the EGF Ca2+ binding domain. The ICD contains a tyrosine kinase phosphorylation region, possibly involved in signaling pathway of ELTD1, for which scant data is available. Besides this, based on the cleavage compartmentation criteria, the ELTD1 receptor consists of an N–terminal Fragment and a C–terminal Fragment [24,32]. The structure is represented in Figure 3.

There are many aspects regarding ELTD1 signaling pathways that have not been clarified, although few important steps forwards have been recently made.

A recent study on cell lines showed that silencing ELTD1 can regulate the endothelial metabolism by suppressing the mitochondrial gene of solute carrier family 25 member 1 (SLC25A1) and the ATP citrate lyase gene (ACLY) [32]. Furthermore, silencing the ELTD1 induced the expression of the hematopoietic stem cell regulator (KIT) [32]. These findings suggest that there is a relationshipbetween ELTD1 and the Notch signaling pathway. The Notch signaling pathway was influenced by suppressing Hes Family BHLH Transcription Factor 2 (HES2) and Jagged Canonical Notch Ligand 1 (JAG1), and by upregulating the Delta Like Canonical Notch Ligand 4 (DLL4) [32]. Additionally, by silencing the ELTD1 in endothelial cells, some components involved in metabolism of pyrimidine, alanine aspartate, glutamine, cysteine, methionine, taurine, arginine, proline, and sugar were found to be upregulated [32]. This study showed non–significant increase in components involved in glycolysis. It is still unclear why ELTD1 silencing leads to ACLY upregulation. This data suggests that regulation of ACLY and SLC25A1 expression by ELTD1 may help to maintain an equilibrium in endothelial metabolism and homeostasis as shown in Figure 3.

More recently, a study conducted by Li et al. showed that JAK/STAT3 signaling pathway (Janus kinases/signal transducer and activator of transcription protein 3) is involved in ELTD1 regulation of proliferation, migration, and invasion of glioma cells. By performing knockdown of ELTD1 in U–87MG and U–138MG cells, they found that the JAK/STAT signaling pathway was inhibited, without effect on other signaling pathways [44]. By using a nude mice orthotopic tumor model, they also found that ELTD1 upregulates the protein expression of HIF–1α (hypoxia–inducible factor 1–alpha), a regulator of tumor formation (cell proliferation, colony formation, migration, and invasion). This data suggests that by ELTD1 silencing, tumor growth could be inhibited and its effect in vivo could be suppressed by HIF–1α overexpression [44] (Figure 3).

Moreover, considering that ELTD1 regulation by the VEGF ligand has been established [24], VEGFR2 association with ELTD1 in glioma was studied using targeted antibody inhibition, proving again that ELTD1 has a key role in angiogenesis, both in vitro and in vivo [18,24].

By treating G55 glioma–bearing mice with either anti–ELTD1 or anti–VEGFR2 antibodies, it was observed that VEGFR2 levels were decreased after anti–ELTD1 antibody treatment, and vice versa, ELTD1 levels were decreased after anti–VEGFR2 antibody treatment, compared to untreated tumors [20]. ELTD1 and VEGFR2 colocalization was also demonstrated by immunohistochemistrystudies. The treatment using anti–ELTD1 antibody significantly increased animal survival, and decreased tumor volumes, compared to IgG–treated or untreated tumor bearing mice [20].

### 3.2. Ligands

Not muchis known about how aGPCRs (in general) and ELTD1 (in particular) are functioning, because of the lack of data on known ligands, receptor activation or its signaling pathways.

One study published by Favara and colleagues specifically investigated ELTD1’s evolution, concluding that its gene appeared cca. 435 million years ago in bony fish and is a highly conserved early core angiogenic gene, with three evolutionary variants [55].

aGPCR signaling is initiated when a tethered agonist binds to a specific extracellular portion of the seven transmembrane helices [56]. Based on that, the conservation mapping of ELTD1 across orthologues was used to hypothesize that its highly conserved external 7TM regions (external loops 2–3, and 4–5) could potentially represent important sites to bind ligands that will initiate the signal transduction of ELTD1. It has been hypothesized that, because ELTD1’s exons are probably an ancestral characteristic, the conservation mapping across vertebrate orthologues could be used to clarify its activation. Furthermore, a functional overlap was detected between ELTD1 and at least one member from a different family, with ability to stimulate angiogenesis based on integrin [57]. Sincethe extracellular matrix is known to have a key role in angiogenesis, it was hypothesized that the extracellular matrix ligands that bind to other aGPCR family members could also bind to ELTD1 [58].

ELTD1 was demonstrated to be regulated by two angiogenic ligands: upregulated by VEGF (vascular endothelial growth factor) and downregulated by DLL4 (Notch ligand delta–like ligand 4) [25,27,31,42], a startingpoint for further investigation.Recently, the Stachelhypothesis has been used to solve the activation mechanism of some orphan aGPCRs [59,60,61]. This hypothesis suggests that, except for GPR123, all aGPCRs express a short 10–20 amino acid tethered agonist called the Stachel peptide, situated C–terminally to the GPS cleavage site, essential for the activation via striking the seven transmembrane receptor loops that initiates signaling [62]. The G–protein–coupled receptor (GPCR) autoproteolysis–inducing domain (GAIN domain) is important for the activity of the GPS cleavage site [63].

Despite all the relevant and productive research work described, the mechanism of ELTD1 activation remains unclarified.

## 4. ELTD1 an Effective Target in a Wide Range of Diseases

Alterations of ELTD1 have been found in several non malignant diseases, but it is also considered to be a potential treatment target in different types of cancers.

For example, the involvement of the ELTD1 receptor has been studied in the following non malignant diseases: multiplesclerosis, schizophrenia, and stroke.

Perturbation in central nervous system (CNS) vasculature is a distinguishing feature in many diseases. ELTD1 antibody therapy was found to affect molecular pathways involved in multiplesclerosis (MS). Towner and colleagues showed that ELTD1 is highly detectable in the brain of mice with experimental autoimmune encephalomyelitis (EAE), as MS model, showing that ELTD1 may represent a promising biomarker for CNS inflammation [64]. In the past, ELTD1 was linked to the cannabis use disorders [50]. Recently, in 2020, based on the symptomatology and psychopharmacology of some CNS disorders, similarities between psychiatric disorders were suggested [65] regarding ELTD1 involvement in schizophrenia development [51].

The identification of some genomic regions, and genes associated with social genetic effects, could represent the basis to better understand the genetic implication for social average daily gain (ADG). By using the genome–wide association strategy in pigs, ELTD1 gene was linked with social genetic effects, suggesting that this receptor could be used as a marker for ADG. Three single nucleotide polymorphisms (SNP) were located upstream the ELTD1 gene, between 161 and 191 kb. Furthermore, it would be of interest to study the association between ELTD1, prostaglandin F2α receptor (PTGFR), and interferon–induced protein 44 (IFI44) genes [66].

A study performed in 2015 focused on African American patients diagnosed with stroke and genome–wide single nucleotide polymorphism (SNP). The study called COMPASS collaboration was the first large–scale GWAS meta–analysis in African Americans individuals with stroke. The data reported showed that the 15q21.3 locus, related with hypertension and high lipid levels, was associated with total stroke. Amongst other, variants of the ELTD1 gene showed nominal associations with various degree of stroke in African Americans individuals [46].

### Malignant Diseases

Between the public health issues worldwide, cancer is one of the most relevant [67]. Studies involving antibodies and small molecules that target specific types of cancer are continuously growing in number, suggesting the importance of this therapeutic approach [68]. These targeted cancer therapies are being studied as single strategies, or in combination with others [69,70,71]. Although it could represent a major step forward in personalized medicine [72], the molecularly targeted therapy has substantial limitations [73], which are motivating the researchers to develop novel approaches based on emerging technologies [74,75].

**Figure 4:**
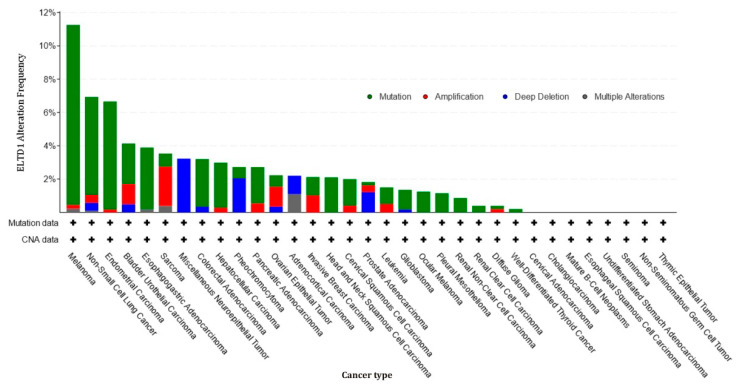
Alteration frequency of ELTD1/ADGRL4 in different types of human cancers. The results were retrieved from the database of BioPortal for Cancer Genomics [76].

By analyzing the alterations of ELTD1 genetic sequence in different cancers usingcBioPortal for cancer genomic database [76], in Figure 4 it can be observed that ELTD1 alterations have a very low frequency in several malignancies such as glioblastoma, ocular melanoma, renal cell carcinoma, colorectal cancer, thyroid cancer, and ovarian cancer, which may suggest that mutation levels are not influencing the tumor evolution, even though high levels of its expression may indicate the presence of the respective malignancy [18,76,77].

Hepatocarcinoma: In a recent study, Kann et al. showed that silencing of ELTD1 drastically reduced hepatocellular carcinoma cells invasiveness [49], confirming previous studies that linked ELTD1 to mechanismsinvolved in the metastatic process [25].

Retinoblastoma: In January 2021, Guihurt Santiago and colleagues reported differences regarding the expression and functional roles of ELTD1 and G–protein receptor 125 (GPR125/ADRGRA3)—two adhesion–GPCRs in retinoblastoma (Rb) [52]. The investigation demonstrated for the first time, that ELTD1, and not GPR125, was overexpressed in Rb compared to fetal retinas. By disrupting ELTD1, in vitro cell migration and in vivo metastasis were reduced without affecting cell viability. This data suggests that ELTD1 may be a potential target for prevention of extraocular Rb and for treatment ofmetastatic Rb [52].

Renal and colorectal cancer: It is well known that ELTD1 and GPR116 are two members of the adhesion G–protein–coupled receptor family expressed in endothelial cells [27]. A study performed by Lu and colleagues focused on their functions using mice lacking ELTD1 and G–protein receptor 116 (GPR116) [78]. The renal and cardiovascular functions were not influenced by the loss of either ELTD1 or GPR116, while the loss of both receptors led to perinatal lethality in half of the mutants, due to cardiovascular malformations (aortic arch arteries and cardiac outflow tract). In addition, the surviving mice showed hemolysis, splenomegaly, and renal thrombotic microangiopathy, with a significant mortality. Meanwhile, the loss of ELTD1 and GPR116 in neural crest–derived cells and endothelial cells did not lead to repetition of any of the phenotypes detected in ELTD1–GPR116 deficient mice, suggesting that loss of these two receptors materialized in cardiovascular and renal defects [78].Common treatment strategies in metastatic renal cell cancer (mRCC) consists in inhibiting the development of new blood vessels by using sunitinib, a tyrosine kinase inhibitor of the vascular endothelial growth factor (VEGF) and platelet–derived growth factor (PDGF) receptors signaling [79]. In 2020, Niinivirta et al. linked the ELTD1 expression level to the progression free survival (PFS) after sunitinib treatment. The expression of ELTD1 in tumor vessels was a positive predictive marker for the sunitinib treatment in patients with renal cancer [22]. A significantly higher PFS after sunitinib treatment was observed in patients with high ELTD1 expression compared to low ELTD1 expression (8 months vs. 5.5 months). On the contrary, the expression level of VEGFR2 had no correlation with sunitinib response. Moreover, this study showed that for sunitinib therapy, ELTD1 may be considered a predictive and not a prognostic marker [22].

Head and neckcancer: In 2013, Massiero et al. analyzed the genes that could potentially be involved in angiogenesis, by profiling the in vivo expression and characterized of the most important candidates using in vitro and in vivo models. By comparing head and neck tumors with normal tissues, a significant increase was observed in ELTD1 expression in tumor–associated cultured endothelial cells (ECs) [18]. Furthermore, increased ELTD1 levels in endothelial cells were also correlated with high microvascular density in head and neck cancers, suggesting its involvement in tumor angiogenesis [18]. Additionally, by profiling the ELTD1 expression in head and neck squamous cell carcinoma, a significant inverse correlation between *CA9ELTD1* (a hypoxia–inducible gene) and ELTD1 mRNA levels was observed [80], probably because of a better perfusion in high ELTD1 tumors [18].

Ovarian cancer: In the same study performed by Massiero and colleagues, primary ovarian human tumor samples were used to study the ELTD1 protein expression. Upregulation of EC ELTD1 expression was observed in neoplastic ovarian tissue, compared to normal tissue. Additionally, higher EC ELTD1 was significantly correlated with increased OS in ovarian tumors [18]. A study performed by Favara et al. showed that ELTD1 was upregulated in several types of cancer cells, including ovarian vascular smooth muscle cells and in tumor–associated endothelial cells, both in zebrafish and humans [55]. By silencing Eltd1 gene in ovarian tumor xenografts in mice, the tumor growth was substantially limited by inhibiting tumor–vessel angiogenesis. In human ovarian cancer patients, increased tumor–vessel endothelial ELTD1 expression was linked to improved OS in patients treated with anticancer therapy. These results show that ELTD1 is a prognostic marker of favorable outcome inhead, neck, and ovarian cancer patients, may be because increased ELTD1 expression could correlate with higher microvessel density, allowing an improved anticancer targeted drug delivery [55].

Glioblastoma:In the beginning of 2013, Towner and colleagues conducted a study proving that ELTD1 expression is a marker for high grade glioma [42]. Few years later, Ziegler et al. used ELTD1 as an antiangiogenic target for treating glioma in mouse and human xenograft glioma models [25]. Furthermore, Serban et al. found that ELTD1 silencing induced cell death in glioblastoma [24,31]. Supporting the above data, Dai S et al. proved that miR–139–5p inhibited tumor progression by targeting ELTD1 [43]. Recently, in vitro and in vivo experiments demonstrated that ELTD1 plays a very important role in proliferation, migration, and invasion of glioma cells, and its overexpression was correlated with poor overall survival (OS) and disease–free survival (DFS) rates in glioma patients. The same study offered evidence that the JAK/STAT3/HIF–1α signaling could control this process [44].

In the last years, ELTD1 was found to be highly expressed in human gliomas, a very aggressive type of brain cancer, and treatments have been started using anti–ELTD1 polyclonal antibodies in glioma preclinical models, but with promiscuous pAb binding [25]. A further study was performed using monoclonal anti–ELTD1 in G55 xenograft glioma mice models, with promising results [29]. The treatment using monoclonal anti–ELTD1 antibody showed high binding specificity, increasing the lifespan, normalizing the vasculature and reducing the tumor volume, compared with the untreated or polyclonal–treated mice. Additionally, a very important result was that ELTD1 interacted and interrupted Notch1 signaling pathway [29]. These data support the idea that ELTD1 may represent a drug target in glioblastoma therapy.

Oligodendroglioma:A network–based strategy was developed to identify novel cancer gene candidates in the region of the 1p/19q codeletion, responsible for some primary human brain tumors, such as oligodendrogliomas. Yet, there is scant evidence regarding the pathology of the above chromosomal mutation. ELTD1, a glioblastoma validated oncogene located on 1p, was predicted to have strong pushing impact on signaling and metabolic pathways involved in oligodendroglioma development [81].

Table 1 summarizes the above information regarding ELTD1 role in malignant diseases.

The preclinical trials targeting ELTD1 are organized in the Table 2.

At present, the use of ELTD1 as therapeutic target in clinical practice has not been studied in large. Even if ELTD1 is of clinical and therapeutic interest, it is the most poorly studied of the GPCR protein families. Relatively littleis known about the receptor intracellular signaling or its activating ligand. Available studies about X–ray crystal structures of ELTD1/ligand complex or ELTD1/intracellular proteins complexes, to validate the binding site on the protein–protein interface, does not practically exist in the literature. In particular, small–molecule inhibitors for ELTD1 have not yet been identified. In a recent study, published in 2021 by MarjutNiinivirta et al., was reported that high expression of ELTD1 in the tumor vasculature predicts a favorable response to sunitinib treatment, in patients with metastatic renal cell cancer [22]. However, no proposed, ongoing or completed clinical trials involving ELTD1 are currently reported in the literature.

## 5. Conclusions

There are many unclear aspects regarding ELTD1 structure, associated ligands, andmechanisms of action. It is well known currently that ELTD1 is highly expressed in tumor endothelial cells in many cancers and recent evidence shows that. Being associated with angiogenesis, it may be a putative predictive biomarker.

All the cited studies support a specific role of ELTD1 in migration and invasion of cancer cells and shed a new light on a new path to better understand the tumor behavior with the hope of identifying and developing new therapeutic strategies in cancer therapy.

However, there are still many questions that need an answer, like:

- Are ELTD1 and other angiogenesis genes reciprocally affected?
- What other ligands may bind to ELTD1 receptor, apart from VEGFR and DLL4?
- What other molecules are involved in the signaling pathways of ELTD1?

Theanswers to these questions still need to be provided and new research fields need to be explored, in order to provide a more complete elucidation of ELTD1 role in malignant diseases, before its introduction into clinics.

## Figures and Tables

**Table 1:** ELDT1 role in cancer.

Type of Cancer	Presumed Role	Observations
Hepatocarcinoma	ELTD1 supports the tumor invasiveness	By silencing of ELTD1, the hepatocellular carcinoma cells invasiveness was drastically reduced [49]
Retinoblastoma	ELTD1 is overexpressed in Rb	ELTD1, was found to be overexpressed in Rb compared to fetal retinas. By disrupting ELTD1, in vitro cell migration and in vivo metastasis were reduced [52]
Renal and Colorectal Cancer	ELTD1 is involved in renal thrombotic microangiopathy and may represent a positive predictive marker after sunitinib treatment	The mice lacking ELTD1 and G–protein receptor 116 (GPR116) showed hemolysis, splenomegaly and renal thrombotic microangiopathy, [78]. A significantly higher PFS after sunitinib treatment was observed in patients with high ELTD1 expression compared to low ELTD1 expression. ELTD1 may be considered a predictive and not a prognostic marker [22]
Head and Neck Cancer	ELTD1 is involved in angiogenesis	Increased ELTD1 levels in endothelial cells were correlated with high microvascular density in head and neck cancers, suggesting its involvement in tumor angiogenesis [18], and also a significant inverse correlation between a the CA9ELTD1 and the ELTD1 mRNA levels was observed [18,80].
Ovarian Cancer	ELTD1 is overexpressed in ovarian cancer	Upregulation of EC ELTD1 expression was observed in neoplastic ovarian tissue, compared to normal tissue. [18,55]. ELTD1 may be a putative prognostic marker with favorable outcome in head, neck and ovarian cancer patients
Glioblastoma	ELTD1 expression is a marker for high grade glioma and a suitable antiangiogenic target	ELTD1 was used as an anti–angiogenic target for treating glioma in mouse and human xenograft glioma models [25,42]. Furthermore, by silencing ELTD1, cellular death was induced in glioblastoma [24,31]. Supporting the above data, miR–139–5p inhibited tumor progression by targeting ELTD1 [43]. In vitro and in vivo experiments showed that ELTD1 has an important role in proliferation, migration, and invasion of glioma cells. There are evidences that the JAK/STAT3/HIF–1α signaling could control this process [44].
Oligodendroglioma	ELTD1 has a strong pushing impact on oligodendroma signaling and metabolic pathways	ELTD1, a glioblastoma validated oncogene located on 1p, was predicted to have strong pushing impact on signaling and metabolic pathways involved in oligodendroglioma development [81].

**Table 2:** ELTD1 preclinical trials.

Preclinical Trial	Observations
ELTD1, an effective antiangiogenic target for gliomas: preclinical assessment in mouse GL261 and human G55 xenograft glioma models	Data regarding tumor volume and OS showed that by using antibodies against ELTD1, glioma growth could be inhibited even more if compared with other therapeutic targets (VEGFR). Untreated GL261 mouses had significantly higher ELTD1 levels compared with mouse normal brain. The therapy involving antibody against ELTD1 had an anti–angiogenic effect observed in microvessel density, magnetic resonance angiography and perfusion measurements, with decreased vascularization compared with controls [25]
ELTD1 as a biomarker for multiple sclerosis: Preclinical molecular–targeted studies in a mouse experimental autoimmune encephalomyelitis model	ELTD1 antibody therapy affected the molecular pathways involved in multiplesclerosis, with a high level of ELTD1 expressionis in the brain of mice experimentally induced with autoimmune encephalomyelitis [64]

## Data Availability

Not applicable.
